# Interpreting the cryo-EM map

**DOI:** 10.1107/S2052252518018304

**Published:** 2019-01-01

**Authors:** Peter B. Rosenthal

**Affiliations:** a Structural Biology of Cells and Viruses Laboratory, The Francis Crick Institute, 1 Midland Road, London, NW1 1AT, UK

**Keywords:** electron cryomicroscopy, signal detection, false discovery rate, cryo-EM map, single-particle analysis, map sharpening, subtomogram averaging

## Abstract

Beckers *et al.* [*IUCrJ* (2019), **6**, 18–33] propose a general approach to visualization of cryo-EM maps. Their method overcomes some of the challenges inherent in the conventional approach for depicting maps using a single isosurface threshold.

Several decades of protein structure determination by X-ray crystallography have produced methods for the calculation of electron density maps of proteins and nucleic acids, and experience in their interpretation by atomic coordinate models. Electron cryomicroscopy (cryo-EM) is now providing new views of biological macromolecules and brings unique requirements for the calculation of maps and their interpretation.

Reconstruction by single-particle analysis has the advantage of recording images containing both amplitude and phase information, so there is no phase problem as in crystallography. In X-ray crystallography, initial maps and phases are improved by iterative model building and refinement. In single-particle analysis, as currently practiced, the calculation of an electron potential map is largely complete before interpretation by atomic models and their refinement begins (Nicholls *et al.*, 2018 ▸). Cryo-EM maps from low to high resolution have been interpreted by models. Increasing map resolution is associated with the appearance of recognizable protein features such as secondary structure (α-helices, β-sheets) and amino acid side chains. In recent years high-resolution maps that show detailed features to 3 Å resolution or better have produced considerable excitement.

The signal in an electron potential map fades with resolution, and as a consequence features at the resolution limit that may be important to accurate model building may be weak and difficult to interpret. Selecting a map threshold for viewing, typically as an isosurface, will determine the features that are observed. The contour may be chosen to match the known molecular volume of its contents. To detect the highest resolution features of interest, a common approach is to simultaneously view several isosurfaces, calculated at multiples of the standard deviation from the mean value of the map, which reflect the varying strength and reliability of features. A new, objective approach to visualization of maps is presented in the paper by Beckers *et al.* in this issue of **IUCrJ** (Beckers *et al.*, 2019 ▸).

The loss of contrast in maps at high resolution that obscures features necessary for interpretation may come from many sources including molecular motion and heterogeneity, imperfect imaging, and incoherent averaging of image data. Contrast loss may be measured from map amplitudes as a function of resolution and modelled by a Gaussian fall-off described by an overall temperature factor or *B* factor. In recent years improvements in imaging and computation have reduced contrast loss. Map contrast may be restored by applying an inverse (negative) temperature factor to enhance high- relative to low-resolution features, a procedure called map sharpening. Sharpening can also amplify noise, and may be applied with a weighting factor (figure-of-merit) to supress noise (Rosenthal & Henderson, 2003 ▸). In current practice, this is part of a map ‘post-processing’ step in single-particle analysis.

Outside the crystalline state, macromolecules are free to adopt different conformations. Key advances will be made in understanding how these states or motions are related to function. However, even a single conformer may be more ordered at its interior than at its periphery as a consequence of motions. Blurring of the map periphery may also result from problems during single-particle averaging. Thus resolution is not uniform across a map, features may differ for a given choice of map threshold, and different degrees of sharpening may be required. A general approach to local map sharpening has been proposed by Jakobi *et al.* and implemented in the program *LocScale* (Jakobi *et al.*, 2017 ▸). Following the construction of an atomic model, map amplitudes are scaled against local amplitudes computed from a model. These have the effect of making maps globally more uniform at a given contour threshold. An approach to automatic map sharpening has also been implemented by Terwilliger *et al.* in which sharpening values are chosen according to map features and their connectivity as measured by map contour surface area, which requires no prior coordinate model (Terwilliger, Sobolev *et al.*, 2018 ▸).

Nevertheless, contouring maps for building and reporting the significance of map features has remained subjective. Beckers *et al.* (2019 ▸) now describe confidence maps, calculated from the electron potential map, combined with an objective thresholding criterion, allowing the assessment of the significance of map features compared to background. They implement a statistical procedure called false discovery rate control, originally devised by Benjamini & Hochberg (1995 ▸). The procedure is broadly applicable in multiple hypothesis testing, and has been applied previously to image and volume thresholding in astronomical and medical imaging.

Confidence maps are thresholded according to the false discovery rate (FDR), which is the single free parameter in the procedure, and displayed as an isosurface. The authors show that when the confidence maps are thresholded with the FDR at 1%, the isosurfaces show structural features remarkably similar to those in electron potential maps (Fig. 1 ▸), but are better at detecting weak features closer to the noise level. An important part of the procedure is the estimation of the background noise level, usually determined by an area of the map that is outside the envelope of the molecule. The procedure is best applied as a post-processing step to an already sharpened map, otherwise the map may lack features, or following resolution filtering (Cardone *et al.*, 2013 ▸) which makes features more uniform across the map.

Applications to a wide range of molecules, including several chosen from the Electron Microscopy Data Bank’s map and model challenges (http://challenges.emdatabank.org), show that weak features in potential maps including side chains and loop density can be supported by the FDR maps to assist in their tracing. These show more uniform display of weak density map features at a single threshold that would otherwise require interrogation by maps calculated at several sigma levels. The confidence maps are fast to calculate, require no prior information from atomic models, and will provide complementary information for use in interpreting electron potential maps. The procedure may also be useful for lower-resolution maps calculated by subtomogram averaging. Ultimately, the community of structural biologists will learn how to best apply them.

There is still a need for tools to validate atomic models and bring them into agreement with electron potential maps and the underlying single-particle image data. Nevertheless, recent advances suggest that many of the steps in building and refinement may be completely automated (Terwilliger, Adams *et al.*, 2018 ▸). We can anticipate improvements in imaging methods and computational image analysis to calculate maps with stronger and more certain features. But then researchers will use their microscopes to image smaller and more heterogeneous specimens. Objective procedures for evaluating map features, such as those proposed by Beckers *et al.*, will concern us for some time to come.

## Figures and Tables

**Figure 1:**
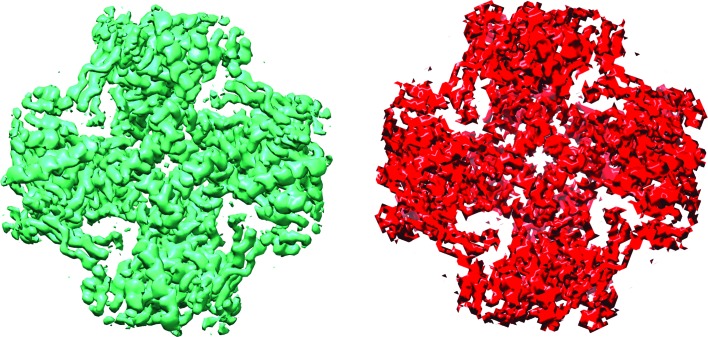
Electron potential map (green) for the TRPV1 channel (EMD-5778) at 3.4 Å (Liao *et al.*, 2013 ▸) and a confidence map (red) calculated by the method described in Beckers *et al.*
